# Applications and Effectiveness of 3D Printing in Various Ankle Surgeries: A Narrative Review

**DOI:** 10.3390/life15030473

**Published:** 2025-03-15

**Authors:** Jeong-Jin Park, Jun Young Choi, Jung-Min Lee, Hyun-Gyu Seok, Chul Hyun Park

**Affiliations:** 1 Department of Orthopedic Surgery, Korea Armed Forces Athletic Corps, Mungyeong 36931, Republic of Korea; wjdwls3912@naver.com; 2 Department of Orthopedic Surgery, Inje University Ilsan Paik Hospital, Juhwa-ro 170, Ilsanseo-gu, Goyang 10380, Republic of Korea; osddr8151@paik.ac.kr; 3 Department of Orthopedic Surgery, Armed Forces Hongcheon Hospital, Hongcheon 25164, Republic of Korea; biomechljm@gmail.com; 4 Department of Orthopedic Surgery, Dongsan Hospital, Keimyung University, Daegu 42601, Republic of Korea; rkaldhkthfl4@naver.com; 5 Department of Orthopedics, College of Medicine, Yeungnam University, Daegu 42415, Republic of Korea

**Keywords:** 3D printing, ankle surgery, implant, instrumentation

## Abstract

Background: Technological advancements have made three-dimensional printing prevalent in orthopedic surgery. It facilitates the production of customized implants and tailored patient instruments, enhancing surgical planning and results. This review focuses on the uses and effectiveness of patient-specific products manufactured using three-dimensional printing in ankle surgery. Methods: A search of databases—PubMed, Embase, Cochrane Library, and Google Scholar—yielded 41 articles for review. Results: Total talus replacement offers a viable alternative to standard treatments like arthrodesis and total ankle arthroplasty. Custom implants and patient-specific instrumentation in total ankle arthroplasty procedures guarantee a tailored fit and accurate alignment. For arthrodesis, three-dimensional printing enables the production of cages, effectively solving issues associated with conventional bone grafts, such as poor bone quality, significant defects, and nonunion. Additionally, patient-specific instrumentation facilitates the swift and accurate placement of Kirschner wires at the correct sites. When performing supramalleolar osteotomy, patient-specific instrumentation leads to shorter operation times, reduced blood loss, and less radiation exposure. Conclusions: Three-dimensional printing is increasingly employed in ankle surgeries, and as technology advances, it is anticipated to become critical for addressing complex ankle issues.

## 1. Introduction

Three-dimensional (3D) printing, also known as additive manufacturing or rapid prototyping, creates objects using materials based on 3D model data [1,2,3]. Although it was developed over 30 years ago, early adoption was hindered by high costs and limited accessibility [4,5]. Nevertheless, recent technological advancements have made 3D printing more affordable and widely available, increasing its application in orthopedic procedures. In orthopedic surgery, a combination of plastic, ceramic, and metal is often used to manufacture customized implants and instruments [6,7]. These individualized designs can be further refined to improve the fit and function of the implant, enhancing performance and promoting better integration with bone, ultimately elevating surgical outcomes [3]. Moreover, the anatomical structure of the bone can be reproduced as a full-size 3D-printed model. Such models assist surgeons in visualizing anatomical features before surgery, enabling precise preoperative planning and surgical simulation with the actual implants [3,8]. Therefore, 3D printing is utilized in various orthopedic applications, such as scoliosis correction, complex intra-articular trauma cases, pediatric deformity correction, arthroplasty, and custom orthotics [3,8].

Total talus replacement (TTR) is the most common use of 3D printing in the ankle joint [5,6,9,10]. Traditionally, conditions affecting the ankle and hindfoot, including arthritis, avascular necrosis (AVN), osteochondral lesions, tumors, and bone infections, are managed through techniques like ankle or tibiotalocalcaneal (TTC) arthrodesis and total ankle arthroplasty (TAA) [5,6]. Arthrodesis frequently results in significant disability in the ankle joint and a risk of nonunion [11]. Although TAA maintains ankle motion, it has encountered failures due to the subsidence and loosening of the talar component [12,13,14]. In contrast, TTR features 3D-printed talar implants customized for each patient and is increasingly recognized as a suitable alternative for treating ankle and hindfoot issues, addressing many of the shortcomings of previous methods [5,6,15,16,17]. Additionally, patient-specific products manufactured using 3D printing are used in various ankle surgeries, such as arthrodesis, TAA, and osteotomies, with encouraging clinical and radiographic results noted [3,18,19,20,21]. Although 3D printing is being used in various fields of ankle surgery, there is still no review article on when and how it is effective. This narrative review examines the various applications of 3D printing in ankle surgery and evaluates the effectiveness of patient-specific products compared to standard implants and instruments.

## 2. Materials and Methods

### 2.1. Literature Search Strategy

The following online databases were explored for articles published until 10 October 2024: PubMed, Embase, Cochrane Library, and Google Scholar. Various combinations of the following search terms were utilized: “three-dimensional,” “three-dimensional print,” “3D,” “3D print,” “additive manufacturing,” “custom,” “patient-specific,” “ankle,” “ankle surgery,” “total talus,” “talar replacement,” “talar prosthesis,” “ankle arthroplasty,” “arthrodesis,” “osteotomy,” “arthroscopy,” “ankle arthritis,” “avascular necrosis,” “trauma,” “tumor,” “infection,” and “osteomyelitis.”

### 2.2. Study Selection

We set the following criteria for selecting articles: (1) studies published in English; (2) quantitative research, including cohort studies, case–control studies, and cross-sectional analyses; (3) case reports; and (4) research that explicitly involves 3D printing in ankle surgery. The exclusion criteria consisted of (1) editorial pieces, letters to the editor, review articles, and book chapters; (2) studies presenting duplicated data; (3) studies lacking a description of the 3D printing production process; (4) studies that did not evaluate clinical or radiographic outcomes following surgery; and (5) studies with unclear postoperative follow-up periods.

### 2.3. Data Collection

We used a three-stage search strategy for this review: title screening, abstract review, and final article selection for full-text analysis. Two authors (J.J.P. and C.H.P.) independently assessed all relevant articles. Duplicate articles were excluded, and the remaining articles were assessed for eligibility based on their titles and abstracts. Articles that could not be retrieved were excluded. After screening, the full texts of eligible articles were independently reviewed by two authors, who reassessed the eligibility of each article. Disagreements were discussed and resolved by reaching a consensus. The co-authors (J.Y.C., J.M.L., and H.G.S.) helped resolve conflicts during discussions.

## 3. Results

### 3.1. Study Selection and Characteristics

**Figure 1:**
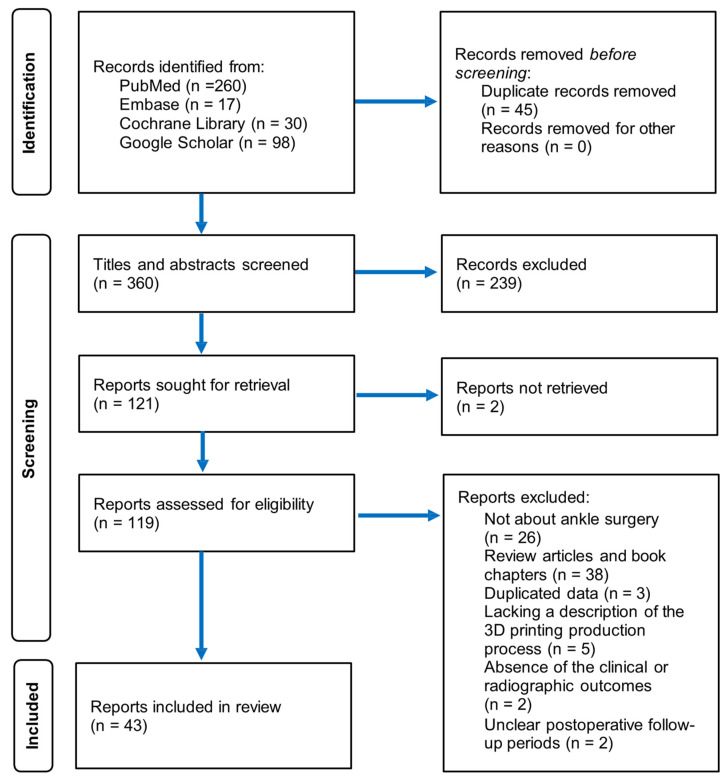
Flow chart depicting selection of articles.

The flow and detailed selection process of the paper review is illustrated in Figure 1. Initially, 360 articles were identified, from which 45 duplicates were removed. A subsequent examination of the titles and abstracts of the remaining articles led to 121 being deemed appropriate for further evaluation. The two articles that could not be retrieved were excluded. A thorough review of the full texts of 119 articles, applying specific inclusion and exclusion criteria, resulted in the exclusion of 76 articles. Ultimately, 43 articles were included in this narrative review. The studies selected were categorized based on the types of ankle surgeries that incorporated 3D printing and were divided into four primary groups: TTR, TAA, arthrodesis, and supramalleolar osteotomy (SMO). This review encompassed 26 studies on TTR, 5 on TAA, 9 on arthrodesis, and 3 on SMO.

### 3.2. Talar Replacement

Out of the 26 studies focused on talar replacement [9,10,13,14,17,22,23,24,25,26,27,28,29,30,31,32,33,34,35,36,37,38,39,40,41,42], 19 were identified as involving solely TTR [9,10,17,25,26,27,28,29,30,31,32,33,34,35,36,37,38,39,41], including 11 retrospective studies and eight case reports. One case report was a study on total talonavicular replacement (TTNR) [24]. Additionally, six studies documented TTR alongside TAA [13,14,22,23,40,42], consisting of four retrospective studies and two case reports. The details of these studies are summarized in Table 1.

Options for treating end-stage talar conditions, including advanced ankle arthritis, osteomyelitis, and talar AVN, are limited [17]. The typical interventions include ankle arthrodesis, TAA, and, in more severe situations, below-knee (BK) amputation [6,25,43]. Although ankle arthrodesis can provide stability for everyday activities, it frequently leads to unsatisfactory clinical outcomes, particularly in bilateral cases, where movements requiring ankle flexion and extension become challenging [44]. Additionally, ankle arthrodesis often involves lengthy recovery periods and carries a risk of complications related to bone healing [44]. TAA presents a viable alternative; however, component loosening, subsidence, and collapse hinder its long-term effectiveness [4,45]. Severe talar injuries, including talar extrusion and comminuted fractures of the talus, as well as unsuccessful TAA, have typically been managed through interventions such as TTC arthrodesis, pantalar arthrodesis, or talectomy [4,5,6]. However, these approaches frequently result in high rates of complications and less-than-ideal functional results, highlighting the necessity for improved treatment methods [26,46]. Recently, TTR has surfaced as an alternative treatment option for these cases [5].

Initial research primarily centered on partial talus prostheses. In 1997, Harnroongroj et al. [47] were the first to report on replacing the talar body with a stainless steel prosthesis, which yielded positive outcomes in 28 out of 33 patients. Conversely, Taniguchi et al. [48] found less promising results, with only 54% of patients achieving good or excellent outcomes using a ceramic talar body prosthesis. Failures in talar body replacement were attributed to loosening of the talar neck or deterioration of the talar head, leading them to conclude that this method was not ideal for managing talar AVN [48]. More recently, Taniguchi et al. analyzed 55 cases of talar AVN treated with a complete total talar replacement (TTR) using a ceramic prosthesis [26]. After an average follow-up period of 52.8 months, the results for the 55 cases showed favorable outcomes in clinical scores and ankle range of motion (ROM). Additionally, the prostheses remained stable without indications of sinking or osteolytic changes in the surrounding bones. These findings mark a transition from partial to total talar replacements.

The present review encompasses 11 retrospective cohort studies [9,10,17,26,32,33,34,35,38,39,41] focusing solely on cases where talar total replacement (TTR) was conducted, extending to the study by Taniguchi et al. Seven of these studies concentrated on talus pathology linked to AVN [9,10,17,26,32,38,39], while one examined severe trauma [34], and another focused on malignant tumors [41]. The remaining two studies addressed talus conditions related to trauma, bone tumors, and AVN [33,35]. Most studies that investigated TTR were centered on talar AVN, whereas those concerning trauma and tumors were less prevalent and reported fewer cases. The notably low occurrence of comminuted talar fractures and malignant tumors likely influenced these findings [33,35]. Except for the study by Joseph et al. [32], which solely analyzed radiographic parameters, all the retrospective studies yielded overall positive postoperative clinical scores and ankle functioning. In the research by Ryuhei et al. [34], which involved patients who underwent TTR due to comminuted talar fractures, two out of six patients faced ongoing restrictions in ankle ROM after surgery. In one instance, the patient was transferred to the authors’ hospital 87 days after injury, contributing to decreased original talus volume and contracture of the ankle’s posterior elements. This resulted in limited dorsiflexion after surgery, necessitating a posterior release. Ryuhei et al. [34] highlighted the significance of preserving talar space through external fixation before surgery to avoid limitations in ankle ROM postoperatively. In a study conducted by Chayanin et al. [33], talar transfer reconstruction (TTR) was performed on two patients with talar loss due to severe trauma, two with giant cell tumors, and one with AVN. This research also involved the use of external fixation for managing open injuries in two trauma patients to sustain talar space. Interestingly, it was observed that those with pre-existing talar tumors or AVN showed better ankle mobility after surgery compared to patients who experienced post-traumatic talar body loss or total talar loss. When considering these two studies [33,34], there appears to be a potential link between TTR after trauma and restricted postoperative ankle ROM. However, the limited number of trauma cases, along with differences in patient characteristics, types of injuries, timing of surgery, and implant materials, complicates the ability to confirm this correlation.

Radiographic results were assessed in all retrospective studies using X-ray or computed tomography (CT) during the follow-up period. Two studies investigated the changes in radiographic parameters following TTR. Joseph et al. [32] found significant improvements in talar height, tilt angle, and Meary’s angle following surgery. Mi Dou et al. [38] noted notable enhancements in talar height and Meary’s angle. These results indicate that TTR has the potential to restore proper joint alignment. Most radiographic assessments concentrated on complications such as implant malposition, prosthesis loosening, and degenerative changes in the joints and bones surrounding the talus, as reviewed in the following nine studies.

Taniguchi et al. [26] analyzed 55 TTR cases for talar AVN with an average follow-up period of 52.8 months, reporting no implant shrinkage or osteolytic changes. Osteosclerosis was observed in 45% of the distal tibia cases, 9% of the navicular cases, and 35% of the calcaneus cases, but these findings did not affect clinical outcomes. Ryuhei et al. [34] examined six TTR cases for comminuted talar fractures over a mean follow-up of 16.4 months, where osteoarthritis (OA) developed on the tibial side in one case, leading to revision surgery to replace the tibial articular surface.

Rishin et al. [9] reviewed 27 TTR cases for talar AVN with a mean follow-up of 22.2 months, finding significant distal tibia AVN in one case that required reoperation. Chayanin et al. [33] looked at five TTR cases for severe talar loss or damage with an average follow-up of 17.8 months; one case required monitoring for mild implant subsidence, and another involved a periprosthetic calcaneus fracture that healed without surgical intervention.

Abramson et al. [35] assessed eight TTR cases involving complex trauma, bone tumors, and AVN over a mean follow-up of 23 months; only minor tibial wear was noted in one case, with no need for additional revision surgery. Mi Dou et al. [38] investigated nine TTR cases for talar AVN over a mean follow-up of 23.17 months, finding no degenerative arthritis or prosthetic dislocation. Lastly, Wenbin et al. [10] evaluated three TTR cases for talar AVN with a mean follow-up of 53 months, reporting no instances of prosthesis loosening or significant degenerative changes. Minor osteophytes formed on the tibial and navicular sides but did not lead to any significant issues during the follow-up period. Morita et al. [39] studied 19 cases of TTR for talar AVN, with an average follow-up of 152 months, reporting no instances of implant subsidence. While signs of degenerative joint disease were observed, they did not impact overall outcomes, and no patients needed revision surgery. Similarly, Xuanhong et al. [41] examined six cases of TTR for malignant tumors of the talus over an average follow-up of 54.8 months, noting no implant failures or revisions. In a review of nine retrospective studies, all total talus prostheses, except for one case of mild implant subsidence, showed stability without any signs of failure, with most studies indicating either no or only mild degenerative changes around the implants. However, aside from Morita et al.’s research [39], there remains a lack of studies with long-term follow-up, resulting in limited insights into implant longevity and the prevalence and prognosis of degenerative changes. Complications that led to revision surgery included two cases of tibial degenerative joint disease, one case of wound dehiscence as reported by Daniel et al. [17], and one case each of superficial peroneal nerve neuroma and superficial infection noted by Rishin et al. [9]. Overall, complications that required revision were minimal across most studies, and there were no cases necessitating implant removal due to infection, indicating a very high success rate for TTR.

The choice of materials for artificial talus implants is vital for ensuring they are compatible with bone [5]. Although alumina ceramic, stainless steel, and cobalt–chrome are commonly utilized, stainless steel is often less favored due to its relatively high wear rate [6]. Most of the retrospective studies analyzed in this review primarily focused on alumina ceramic and cobalt–chrome, except the research conducted by Chayanin et al. [33], which involved stainless steel. Recently, a variety of material combinations have been investigated. Titanium nitride has gained prominence for its effectiveness in creating joints compatible with natural articular cartilage, as evidenced by studies from Rishin et al. [9] and Mi Dou et al. [38]. Furthermore, Xuanhong et al. [41] used a modular prosthesis of ultra-high-molecular-weight polyethylene and titanium to minimize cartilage wear and facilitate revision procedures. Additionally, Wenbio et al. [10] employed vitallium alloy, known for its superior strength, biocompatibility, and corrosion resistance compared to cobalt–chrome.

The present review encompasses eight reports that focused solely on TTR procedures [25,27,28,29,30,31,36,37]. Yukari et al. [27] and Ichiro et al. [29] conducted TTR to address idiopathic talar necrosis, noting significant improvements in clinical results and favorable radiographic findings at the two-year follow-up. Xiang et al. [30] and Jihui et al. [37] each documented cases of TTR performed after en bloc resection for malignant talus tumors. Xiang et al. [30] assessed the Musculoskeletal Tumor Society score (MSTS), the American Orthopedic Foot and Ankle Society (AOFAS) score, and ankle ROM at the six-month mark, revealing positive clinical outcomes. Jihui et al. [37] conducted evaluations using MSTS and Toronto extremity salvage scores over a one-year follow-up, reporting commendable results and confirming that the patient could walk unaided by the two-year follow-up. Neither study reported any abnormal findings in radiographs throughout the follow-up period.

Kuldeep et al. [25] described a case involving total talar extrusion with a large, contaminated laceration resulting from a vehicle accident; after initially aligning the talus and treating the open wound, an infection developed, necessitating a talectomy. Infection was managed using a cement spacer and a vacuum-assisted closure device, followed by skin grafting. TTR was performed seven months after injury, and by the eleven-year follow-up, the patient exhibited favorable clinical outcomes, with an AOFAS score of 75 and a sagittal ankle ROM of 25°. Radiographs indicated slight plantarflexion of the implant, but it remained stable.

Sebastien et al. [28] reported a case of total talar extrusion where the talus was overlooked at the time of injury, resulting in the immediate application of a non-anatomical cement spacer. Six months later, TTR was executed, and by the two-year follow-up, the clinical and radiographic outcomes were excellent. Shin et al. [31] presented a similar case of traumatic talar loss but managed infection with a non-anatomical cement spacer. Before the final surgery, there was an extended period of using an antibiotic-loaded cement spacer, followed by the placement of a 3D-printed anatomical talus cement spacer. At the 14-month follow-up, the patient was free of infection, could walk for approximately 15 min with mild discomfort, and showed some ankle ROM, with no fractures observed in the cement spacer.

Kimberly et al. [36] described two cases involving an anatomical talus cement spacer. The first case featured a patient with nonunion of a talar neck fracture and resulting post-traumatic ankle arthrosis, who underwent TTR with a cobalt–chrome prosthesis. This initially failed due to infection, leading to the use of an anatomical talus cement spacer. The second case involved a patient with an infected nonunion after subtalar arthrodesis. A talectomy, thorough irrigation, and debridement were conducted, followed by anatomical talus cement spacer application. Follow-ups at 11 months and 4 months showed that the infection had resolved. While the patients had mild pain, both could bear weight in regular footwear and exhibited limited ankle ROM.

Overall, case reports highlight excellent outcomes for TTR in instances of idiopathic necrosis, malignant tumors, and severe trauma. Kuldeep et al. [25] demonstrated that a total talar prosthesis can be effectively maintained without complications over an extensive 11-year follow-up period. Furthermore, the findings from Shin et al. [31] and Kimberly et al. [36] indicate that an anatomical talus cement spacer provides effective antibiotic dispersion for managing infections, can endure compressive loading during weight-bearing activity, stabilizes the ankle joint when the talus is absent, and affords a limited range of ankle motion.

There was one case report of TTNR in which both the talus and navicular were replaced. Giannini et al. [24] reported on a 27-year-old male professional rock climber who developed post-traumatic ankle and talonavicular arthritis due to a complex talar and navicular fracture. In this patient, two standard treatment options were available: ankle and talonavicular arthrodesis, and total ankle replacement combined with talonavicular arthrodesis. However, the most suitable option to meet both the high functional demands and long-term implant survival in a young athlete was TTNR. Giannini et al. [24] performed TTNR using a customized implant manufactured with 3D printing and reported satisfactory functional and radiographic outcomes at the 30-month follow-up. Belvedere et al. [49] assessed the restoration of joint function in this patient at the 30-month follow-up. Gait analysis was performed using stereophotogrammetric, dynamometric, electromyographic, and baropodometric systems, revealing good restoration of rotation and moment patterns in the major lower limb and foot joints of the operated leg. Additionally, videofluoroscopic analysis of the artificial tibiotalar joint showed a flexion capability of approximately 20 degrees, with some motion observed in the frontal and transverse planes. TTNR with a custom-made implant is a valuable clinical option that can serve as an alternative to conventional methods in young, active patients with OA of the ankle and talonavicular joints.

The current review includes six studies on TTR in ankle arthroplasty [13,14,22,23,40,42], with three focusing on failed total ankle arthroplasty (TAA) cases [22,40,42]. Some authors suggest using TTC arthrodesis with a retrograde nail to manage failed TAA alongside talar subsidence and bone loss, which yields high fusion rates and stability [50]. However, this method results in limb shortening, impaired shock absorption, reduced propulsion, and difficulties adapting to uneven terrains [22]. Given these risks, including infection and nonunion, TTR has emerged as a preferable alternative to preserve limb length, alleviate pain, and restore function while upholding biomechanical stability [6]. Shinji et al. [22] reported the first case of TTR as a salvage procedure for TAA failure due to talar body collapse. At the two-year follow-up, the patient resumed daily activities without pain, exhibiting an ankle ROM of 15 degrees in dorsiflexion (DF) and 10 degrees in plantarflexion (PF). The Japanese Society for Surgery of the Foot’s rheumatoid arthritis foot and ankle scale showed a commendable clinical score of 65, and radiographs confirmed well-maintained alignment without signs of loosening. In a prospective study by Strand et al. [40], two patients who received TTR due to failed TAA demonstrated satisfactory outcomes in ankle ROM, AOFAS scores, visual analog scale (VAS) scores, and Short Form-36 Health Survey (SF-36) scores at follow-ups of 24 months and 16 months, respectively. Additionally, a retrospective study by Wang et al. [42] involving 19 patients indicated notable clinical improvements alongside preserved postoperative radiographic alignment following TTR due to failed TAA.

Two retrospective studies [13,14] examined end-stage ankle OA patients exhibiting severe talar collapse. Traditional TAA with surface replacement poses a high failure risk in these severe cases due to subpar bone quality. Kanzaki et al. [13] observed marked enhancements in ankle ROM and JSSF scale scores at a 34.9-month follow-up in 22 combined TAA and total talus prosthesis cases. While complications such as delayed wound healing and medial malleolar fractures were reported, no implant-related issues occurred. Kurokawa et al. [14] conducted a retrospective comparison between standard TAA and combined TAA, revealing better clinical outcomes with the combined approach to TAA.

The last study performed TAA using a total talus prosthesis to preserve ankle function in a young patient with severe talus dislocation, showing satisfactory clinical and radiographic outcomes at the 28-month follow-up [23].

### 3.3. Total Ankle Arthroplasty

This section includes five studies focused on 3D printing in TAA [51,52,53,54,55], four retrospective studies, and one case report. Table 2 summarizes these studies.

While TAA is increasingly replacing arthrodesis, it has led to high levels of patient dissatisfaction and failure [56]. Being a small joint, the ankle represents a limited market, translating to less attention from clinical, industrial, and scientific sectors [46]. This lack of interest has resulted in a narrow selection of prosthetic components, often causing poor fit, significant bone resection, and compromised bone integrity [46]. For effective TAA results, accurate implant positioning is essential since even slight misalignments can greatly affect joint kinematics and contact pressure, ultimately leading to implant failure [12]. Customized implants and patient-specific instrumentation (PSI) utilizing 3D printing have been developed to overcome these issues. Belvedere et al. [51] were pioneers in demonstrating the potential of customized implants in the ankle, conducting their study on three cadaveric normal ankle joints. They reported mean manufacturing errors below 0.08 mm, reflecting the high accuracy of the 3D-printed implants. They observed consistent patterns in manually performed ankle motion, with a mean standard deviation of less than 1.0 degrees, validating good mobility. Additionally, load/displacement curves revealed that the replacements exhibited stability similar to the original ankles.

Faldini et al. [53] were the first to present clinical outcomes from a fully customized TAA case using PSI and a customized implant. They developed a PSI specifically designed to fit the frontal bone of the ankle, detailing the placement of the Kirschner wire, bone cuts, and drill levels. The final implant size and positioning aligned with the preoperative plan, and radiographs confirmed proper implant positioning and alignment. At the 4-month follow-up, the results were evaluated as excellent using VAS, AOFAS, and SF-36 metrics. Gait analysis also indicated satisfactory joint moments at the ankle complex and normal muscle activation timing. Gross et al. [55] assessed the effectiveness of the 3D-printed fourth-generation total ankle in a multicenter prospective study comprising 91 cases utilizing patient-reported outcome measures (PROMs). After one year, improvements were noted in all PROM domains, and the study indicated a low revision rate, reflecting better results than traditional ankle replacements. Doty et al. [54] conducted a retrospective study involving 30 cases of the fourth-generation total ankle prosthesis printed in 3D. Each patient had at least two years of follow-up, averaging 26 months, revealing significant enhancements in Patient-Reported Outcomes Measurement Information System (PROMIS) physical scores and VAS outcomes. The implant demonstrated a notable survival rate of 90%.

Additionally, a study focused on PSI aimed to precisely align the rotation rather than just the coronal and sagittal positions. Gagne et al. [52] carried out a prospective study on 22 TAA cases to evaluate the accuracy of axial rotation utilizing patient-specific guides. In this research, a PSI was created and printed based on a CT scan plan to bisect the medial and lateral gutters accurately. The study compared intraoperative images of axial rotation achieved with the PSI guide against the standard medial gutter fork referencing technique. Using patient-specific guides, reliable positioning of the tibial implant was accomplished in over half of the cases, aligning with the earlier results from the standard medial gutter fork method referencing.

### 3.4. Arthrodesis

This section includes nine studies examining the use of 3D printing in arthrodesis, including seven retrospective studies and two case reports. Table 3 summarizes these studies.

Among the nine studies that explored 3D printing in arthrodesis, six involved TTC arthrodesis [57,58,59,60,61,62], two focused on ankle arthrodesis [63,64], and one dealt with subtalar arthrodesis [65]. All studies on TTC arthrodesis utilized 3D printing to develop custom implants. Salvaging the lower extremity in cases of significant bone defects, compromised bone quality, or nonunion poses challenges [66]. Traditional methods—such as autografts, bulk allografts, vascularized fibular transfer, and commercial spacers—strive to restore limb length and stability but often result in complications like nonunion, graft collapse, and the need for additional surgeries [67,68]. Recently, patient-specific 3D-printed titanium implants have emerged as a promising alternative. These implants offer a tailored fit, enhanced osseointegration, and decreased donor-site morbidity, potentially allowing for lasting, single-stage correction without the recurrent complications associated with conventional techniques [57].

Notably, Hsu et al. [57] reporting a pioneering case that utilized a 3D-printed implant with TTC arthrodesis. A patient-specific titanium truss cage addressed a large bony defect in a patient with persistent distal tibial nonunion. At the one-year follow-up, the patient reported minimal pain and could ambulate without assistance, with radiographs showing well-maintained fusion and satisfactory alignment. Lorena et al. [59] performed TTC arthrodesis with customized 3D-printed titanium scaffolds in seven patients suffering from ankle osteoarthritis (OA) and segmental bone defects. Excluding one patient who required a BK amputation due to recurrent chronic osteomyelitis, six patients achieved complete radiographic union, while one demonstrated partial union at the one-year follow-up. There was a notable improvement in the AOFAS and VAS clinical scores after an average follow-up of 21 months. Steele et al. [58] executed a retrospective comparison involving eight cases with a 3D spherical implant and seven using a femoral head allograft for patients with severe ankle bone defects necessitating TTC arthrodesis. The total fused articulation rate was significantly higher in the 3D implant group, with more patients successfully achieving fusion in all three articulations compared to the femoral head allograft group, which also exhibited notably greater graft resorption. This confirms that the 3D implant group achieved more successful fusion than traditional allograft methods. Additionally, various other studies have reported favorable clinical and radiographic results with the use of 3D-printed implants in conjunction with TTC arthrodesis for managing bony defects in conditions such as end-stage talar AVN, failed ankle arthrodesis, and Charcot disease arthropathy [60,61,62]. Liang et al. [64] conducted ankle arthrodesis using a 3D-printed cage in 13 patients who needed extensive or partial resections due to distal tibia tumors. Similar to earlier studies on TTC arthrodesis, a 3D-printed megaprosthesis was employed to improve mechanical strength, joint stability, and osseointegration compared to conventional techniques. During an average follow-up of 28 months, clinical scores were positive, with only one patient developing a periprosthetic infection from paronychia at the two-year mark.

Duan et al. published two studies utilizing PSI, where 3D-printed personalized guides were designed for arthroscopic ankle and subtalar joint arthrodesis [63,65]. The authors compared outcomes between surgery with these tailored guides and procedures using traditional methods. Arthroscopic ankle arthrodesis is increasingly favored for its minimally invasive nature, higher fusion rates, and lower complication rates [69]. This method generally employs cannulated screws, requiring the precise placement of Kirschner wires to ensure effective screw fixation and successful fusion [70]. The surgeon’s skill primarily influences wire positioning and is frequently verified using C-arm fluoroscopy, which may prolong surgery and increase radiation exposure for both the surgeon and the patient [70]. Duan et al. [63] reported a significantly quicker time for the appropriate placement of Kirschner wires in the PSI group than in the control group, with no meaningful difference in clinical outcomes. This indicates that PSI could effectively minimize surgery time and intraoperative radiation exposure. Like arthroscopic ankle arthrodesis, precise positioning of Kirschner wires is crucial for successful fixation in subtalar arthrodesis. In Duan et al.’s research on subtalar arthrodesis [65], using a 3D-printed personalized guide significantly reduced the time required to accurately drill the Kirschner wire and decreased the need for additional adjustments.

### 3.5. Supramalleolar Osteotomy

This section includes three retrospective studies on the application of 3D printing in SMO [71,72,73]. Table 4 summarizes their characteristics.

SMO benefits mid-stage asymmetric ankle osteoarthritis, but traditional methods often yield less-than-optimal results [74]. This is primarily due to reliance on freehand planning, varying surgeon skills, and fluoroscopy, which can introduce errors [75]. Furthermore, the necessity for repeated fluoroscopic adjustments during surgery can result in additional bone loss, longer operation times, increased intraoperative blood loss, and a heightened risk of complications [76,77]. Recent research indicates that using 3D-printed PSI in SMO improves surgical accuracy and alignment and minimizes surgical difficulties. Faict et al. [71] performed SMO with patient-specific dome-shaped osteotomy guides made from weight-bearing CT scans (WBCT) in five patients. WBCT performed three months after surgery showed healing at the osteotomy site and radiographic improvements in the tibial anterior surface angle (TAS), tibiotalar angle, and hindfoot angle. All patients exhibited positive clinical outcomes after an average follow-up of 40.8 months. Wang et al. [73] executed a retrospective analysis comparing SMO with and without PSI. The PSI group received a patient-specific osteotomy guide plate manufactured by 3D printing. The PSI group experienced shorter average operating times, shorter postoperative hospital stays, fewer fluoroscopic tests, and less reduction in albumin levels. However, the PSI group had a longer preoperative hospital stay for planning and incurred higher costs. While both cohorts showed significant radiologic improvements, the PSI group had more marked enhancements in the TAS and the tibiotalar tilt angle. After a follow-up period averaging 33.4 months, clinical scores, ankle ROM, and improvements in the Takakura stage were superior in the PSI group. Similarly, Zhang et al. [72] found that the PSI group receiving a patient-specific guide exhibited significantly lower operation times, intraoperative blood loss, and intraoperative fluoroscopy duration than the conventional group. Zhang et al. [72] also evaluated the anticipated bone defect size in the PSI group before surgery to manufacture personalized porous tantalum implants, which replaced autologous bone grafts and resulted in favorable clinical results.

### 3.6. Materials

Cobalt–chrome alloys, alumina ceramics, titanium alloys, and vitallium alloys are widely used materials in orthopedic 3D printing, each with distinct mechanical properties, biocompatibility, and long-term stability [78,79]. Cobalt–chrome alloys and vitallium alloys offer high strength with excellent wear and corrosion resistance, making them suitable for load-bearing implants, though potential metal ion release may raise biocompatibility concerns [80]. Alumina ceramics exhibit exceptional hardness, wear resistance, and biocompatibility, but their brittleness increases the risk of fracture [79,80]. Titanium alloys are favored for their high strength-to-weight ratio, superior fatigue resistance, and excellent biocompatibility, promoting strong osseointegration, though concerns exist regarding aluminum and vanadium ion release [79,80]. The above materials are applied in various cases of ankle surgery, and overall, material selection depends on balancing mechanical performance, biological response, and long-term durability for specific implant applications [81].

## 4. Summary

In recent years, surgical treatment trends for end-stage ankle OA have shifted toward TAA, aiming to preserve joint mobility and improve patient quality of life [82,83]. Successful TAA requires adequate bone stability and sufficient bone stock for implant fixation [82]. However, severe bone loss due to AVN, trauma, infection, or tumors can compromise the structural integrity of the talus, making TAA challenging or even contraindicated [82]. In these cases, patient-specific implants and instruments utilizing 3D printing have been successfully used to preserve the ankle joint. TTR has become a promising option for treating severe talar conditions like advanced arthritis, AVN, severe trauma, and tumors, with studies showing favorable clinical and radiographic results. Post-surgical ROM may be restricted in trauma cases, likely due to the complexity of injuries and delays in treatment. Research indicates that TTR achieves stable prosthesis positioning, minimizes degenerative changes in surrounding joints, and restores alignment with low complication rates. Furthermore, antibiotic-loaded anatomical talus cement spacers have proven effective in managing infections in trauma cases, supporting joint stability and allowing for limited ROM during recovery. TTR, combined with ankle arthroplasty, has been utilized to tackle intricate situations of failed TAA and severe talar collapse linked to end-stage ankle OA. While TTC arthrodesis with a retrograde nail offers stability, it may cause limb shortening, decreased shock absorption, and limited adaptability on uneven surfaces. A combined TAA approach serves as an alternative that preserves limb length, alleviates pain, and upholds biomechanical stability. The introduction of 3D printing in TAA addresses issues seen with traditional implants, such as inadequate fit, extensive bone removal, and improper positioning, which can result in high failure rates and patient dissatisfaction. Tailored implants and PSI facilitate precise alignment and a customized fit, which are vital for joint stability and optimal function.

Three-dimensional printing has proven to be a crucial asset in arthrodesis, particularly for complex cases involving large bone defects, poor bone quality, or nonunion. Customized implants ensure a bespoke fit, enhance osseointegration, and decrease donor-site complications, enabling lasting, single-stage corrections. Additionally, 3D-printed surgical guides enhance the accuracy and speed of Kirschner wire positioning, showing the potential to shorten operative time and reduce radiation exposure in arthrodesis. This highlights the value of 3D printing technology in advancing surgical precision and efficiency.

In SMO, 3D printing enhances precision, alignment, and surgical effectiveness, overcoming challenges inherent in traditional methods that heavily depend on freehand planning and fluoroscopy. The use of PSI in SMO has resulted in reduced operative durations, less intraoperative blood loss, fewer fluoroscopy exposures, and improved clinical and radiographic outcomes.

As shown above, patient-specific implants and instruments utilizing 3D printing have been effectively used in various fields of ankle surgery. However, there are generally some issues in the clinical application of 3D printing technology. First, the high initial cost of equipment and production, along with the inability to mass produce, leads to differences in accessibility among medical institutions [84]. Second, the process requires CT/magnetic resonance imaging-based design, printing, processing, and sterilization, resulting in prolonged preparation time, making it unsuitable for emergency surgeries [85,86]. Third, printing precision and anatomical variation among patients may lead to design errors, increasing the risk of PSI misalignment during surgery [84,86]. Lastly, regulatory approval processes vary by country, and issues related to liability in cases of PSI failure and patient data protection must be addressed [84]. Three-dimensional printing has certain drawbacks, including high costs, production time, accuracy issues, and legal regulations. Therefore, it should be carefully utilized in planned surgeries such as joint replacement, tumor surgery, and post-traumatic deformity correction rather than in emergency procedures. With advancements in technology and the resolution of ethical concerns, its applications may further expand in the future.

This review has several limitations. While many studies have shown positive results regarding 3D printing in ankle surgeries, most evidence is derived from case reports and a limited number of retrospective cohort studies. As 3D printing technology advances and costs decrease, its application is anticipated to broaden, allowing for studies involving larger populations. Furthermore, because most studies thus far have concentrated on short-term follow-ups, additional research with statistically validated long-term evidence is necessary.

## 5. Conclusions

Three-dimensional printing is used across various ankle surgeries, such as TTR, TAA, arthrodesis, and SMO. Tailored implants and PSI are progressively adopted as replacements for traditional surgical methods or to improve the accuracy and efficacy of current practices. With ongoing advancements in 3D printing technology and its growing integration into medicine, it is expected that 3D printing will become an increasingly essential resource for addressing intricate and severe ankle issues and pathologies.

## Figures and Tables

**Table 1:** Literature review of articles on 3D printing applications in total talar replacement.

Author	Published Year	Study Design	No. of Cases	Ankle Pathology	Surgery Type	Outcomes	Mean Follow-Up Period (Months)	3D Printing Use (Material)
Kuldeep et al. [25]	2013	Case report	1	Open talar extrusion	TTR	AOFAS score (75), ankle ROM (25°), radiographs (prosthesis was in a slightly PF but stable position)	132	Customized implant (cobalt–chrome)
Taniguchi et al. [26]	2015	Retrospective	55	Talar AVN	TTR	JSSF score (improved from 43.1 to 89.4), AOS (score for “pain at its worst” improved from a mean of 6.1 to 2.0), improved ankle DF	52.8	Customized implant (alumina ceramic)
Yukari et al. [27]	2015	Case report	1	Idiopathic necrosis of the talus	TTR	AOFAS score (improved from 45 to 90), ankle ROM (20° of DF and 40° of PF), radiographs (stable position in ankle mortise, no degenerative or destructive changes in the surrounding bones)	24	Customized implant (alumina ceramic)
Sebastient et al. [28]	2017	Case report	1	Total dislocation	TTR	AOFAS score (improved from 11 to 77), SF-36 (improved from 17 to 82), ankle ROM (10° of DF and 40° of PF), radiographs (no periprosthetic delineation and no premature loosening of the implant)	24	Customized implant (cobalt–chrome)
Ichiro et al. [29]	2017	Case report	2	Idiopathic necrosis of the talus	TTR	JSSF score (improved from 22 and 29 to 90 and 95, respectively), VAS (improved from 9 and 8 to 0 and 0), ankle ROM (10° of DF and 30° of PF, respectively), radiographs (prosthesis was appropriately positioned in the ankle with no degenerative or destructive changes in the surrounding bones)	24	Customized implant (alumina ceramic)
Joseph et al. [32]	2018	Retrospective	14	Talar AVN	TTR	Radiographic parameters (talar height and talar tilt angle were significantly improved, Meary’s angle correction was observed in cavus and planus foot deformity)	5	Customized implant (cobalt–chrome)
Xiang et al. [30]	2018	Case report	1	Mesenchymal sarcoma of the talus	TTR	MSTS score (26), AOFAS score (91), ankle ROM (10° of DF, 30° of PF, 5° of eversion, and 10° of inversion), radiographs (prosthesis and the screws were in a stable position, no abnormalities were observed in the surrounding bones)	6	Customized implant (UHMWPE, titanium alloy)
Daniel et al. [17]	2019	Retrospective	15	Talar AVN	TTR	FAOS and VAS (significant improvements), coronal and sagittal alignment on weight-bearing radiographs (no significant difference)	12.8	Customized implant (cobalt–chrome)
Shin et al. [31]	2019	Case report	1	Traumatic loss of the talus	TTR	Walk independently with mild pain for 15 min with a crutch occasionally, ankle ROM (5° of DF, 10° of PF), radiographs (no visible erosion of the left tibia, navicular, or calcaneus, no fracture of the talus cement spacer)	14	Customized implant (antibiotic-loaded cement spacer)
Ryuhei et al. [34]	2019	Retrospective	6	Comminuted talar dome fracture or talar body defects	TTR	AOFAS score (78.8), ankle ROM (10° of DF, 31° of PF)	16.4	Customized implant (alumina ceramic)
Jihui et al. [37]	2020	Case report	1	Osteoblastic osteosarcoma of the talus	TTR	MSTS (93%), TESS (93), walk normally without support	24	Customized implant (titanium)
Rishin et al. [9]	2020	Retrospective	27	Talar AVN	TTR	FAOS concerning pain, symptoms, and quality of life and VAS (significant improvements), ankle ROM (insignificantly improved), 3 complications requiring reoperation	22.2	Customized implant (cobalt–chromium or cobalt–chromium with titanium nitride coating)
Chayanin et al. [33]	2020	Retrospective	5	Severe talar loss or damage	TTR	VAS (82.3), SF-36 (83.38), mild subsidence (1 patient), periprosthetic fracture (1 patient, a mild displaced calcaneal fracture)	17.8	Customized implant (four cases of stainless steel, one case of titanium)
Abramson et al. [35]	2021	Retrospective	8	2 (complex, irreparable trauma), 4 (post-traumatic AVN with symptomatic collapse), 2 (primary bone neoplasms)	TTR	AOFAS score (79.25), SF-36 (83.25), no revision surgeries, radiological changes in minor tibial wear (1 patient, symptom-free)	23	Customized implant (cobalt–chrome)
Mi dou et al. [38]	2021	Retrospective	9	Talar AVN	TTR	AOFAS scores and VAS (significant improvements), ankle ROM (insignificantly improved), radiographs (no degenerative arthritis or prosthetic dislocation; talar prosthesis was placed in the original anatomical position), radiographic parameters (talar height and Meary’s angle were significantly improved)	23.17	Customized implant (titanium alloy for talar structure, cobalt–chromium–molybdenum alloy for articular facet)
Kimberly et al. [36]	2021	Case report	2	Talus fracture nonunion (failed TTR), infected subtalar arthrodesis nonunion	TTR	WB as tolerated in a regular shoe, 3/10 daily pain in both patients, ankle ROM (10° and 6° of DF, 25° and 20° of PF, 15° and 10° of eversion, and 15° and 10° of inversion, respectively)	11, 4	Customized implant (antibiotic-loaded cement spacer)
Wenbin et al. [10]	2022	Retrospective	3	Talar AVN	TTR	AOFAS score (88.5), radiographs (no signs of prosthesis loosening or serious degenerative change in the surrounding area of the joint, small osteophytes on the tibial side and navicular side)	53	Customized implant (vitallium alloy)
Morita et al. [39]	2022	Retrospective	19	Talar AVN	TTR	AOS and JSSF score (significantly improved), median postoperative ankle ROM (45°)	152	Customized implant (alumina ceramic)
Xuanhong et al. [41]	2024	Retrospective	6	Malignant tumor of the talus	TTR	MSTS-93 score (26.8), AOFAS score (88.5), ankle ROM (9.2° of DF, 32.5° of PF), radiographs (no aseptic loosening, fracture/dislocation of the prosthesis, or screws loosening)	54.8	Customized implant (UHMWPE part and titanium alloy part)
Giannini et al. [24]	2015	Case report	1	Severe osteoarthritis of both the ankle and the talonavicular joints, secondary to talar AVN	Total talonavicular replacement	VAS (1), AOFAS score (81), Tegner activity (9), ankle ROM (11° of DF, 7° of PF), radiographs (no signs of radiolucency around the implant)	30	Customized implant (cobalt–chrome)
Magnan et al. [23]	2004	Case report	1	Open total medial dislocation of the talus	TAA plus total talus prosthesis	No pain or limp during normal activities, ankle ROM (5° of DF, 30° of PF), radiographs (good alignment of both component, no loosening or periprosthetic radiolucent line)	28	Customized implant (titanium)
Shinji et al. [22]	2010	Case report	1	Failed TAA	TAA plus total talus prosthesis	JSSF score (65), no pain during normal activities, and was able to walk without aids, ankle ROM (15° of DF, 10° of PF), radiographs (no evidence of loosening)	24	Customized implant (alumina ceramic)
Kurokawa et al. [14]	2018	Retrospective	10 (combined), 12 (standard)	Ankle arthritis with severe talar collapse	TAA plus total talus prosthesis	JSSF score (postoperative score was significantly higher in the combined TAA group), AOS (no significant differences between the two groups in terms of pre-and postoperative function and postoperative pain)	58 (combined), 64 (standard)	Customized implant (alumina ceramic)
Kanzaki et al. [13]	2019	Retrospective	22	Ankle arthritis with severe talar collapse	TAA plus total talus prosthesis	JSSF score and SAFE-Q score (significantly improved), ankle ROM (significantly improved)	34.9	Customized implant (alumina ceramic)
Strand et al. [40]	2022	Prospective	2	Failed TAA	TAA plus total talus prosthesis	AOFAS score, VAS, and SF-36 improved postoperatively, improved ankle ROM, radiographs (no evidence of loosening or peri-cystic changes)	24	Customized implant (cobalt–chrome)
Wang et al. [42]	2024	Retrospective	19	Failed TAA	TAA plus total talus prosthesis	PROMIS (all domains significantly improved)	37.9	Customized implant (titanium)

TTR, total talar replacement; AOFAS, American Orthopedic Foot and Ankle Society; ROM, range of motion; PF, plantarflexion; AVN, avascular necrosis; JSSF, Japanese Society for Surgery of the Foot; AOS, ankle osteoarthritis scale; DF, dorsiflexion; SF-36, Short Form-36 Health Survey; VAS, visual analog scale; MSTS, Musculoskeletal Tumor Society score; UHMWPE, ultra-high-molecular-weight polyethylene; FAOS, foot and ankle outcome score; TESS, Toronto extremity salvage score; WB, weight bearing; TAA, total ankle arthroplasty; SAFE-Q, self-administered foot evaluation questionnaire; PROMIS, Patient-Reported Outcomes Measurement Information System.

**Table 2:** Literature review of articles on 3D printing in total ankle arthroplasty.

Author	Published Year	Study Design	No. of Cases	Ankle Pathology	Surgery Type	Outcomes	Mean Follow-Up Period (Months)	3D Printing Use
Belvedere et al. [51]	2018	Cadaveric study	3	Normal ankle joint	TAA	Mean manufacturing errors (smaller than 0.08 mm), consistent ankle motion patterns, mobility, and stability (compared well with the original natural conditions)	N-S	Customized implant
Gagne et al. [52]	2019	Prospective	22	Ankle OA	TAA	Compared to the PSI and conventional methods (average difference was −0.46), 50% (11/22) were within 2 degrees of the target, and 77% (17/22) were within 4 degrees of the target	N-S	PSI (guide)
Faldini et al. [53]	2020	Case report	1	Ankle OA	TAA	Clinical abilities (restored without pain), VAS, AOFAS score, SF-36 (improved), gait analysis (quasi-physiological pattern of rotation, normal muscle activation time), radiographs (stable prosthesis with no signs of radiolucency around the implant)	4	Customized implant and PSI (guide)
Gross et al. [55]	2024	Prospective (multicenter)	91	Ankle OA	TAA	Improved in all PROM domains (AOS, PROMIS Global Physical Heath, FAOS symptom scores)	12	Customized implant
Doty et al. [54]	2024	Retrospective	30	Ankle OA	TAA	VAS, PROMIS physical scores (improved), high implant survival rate (90%)	26	Customized implant

TAA, total ankle arthroplasty; OA, osteoarthritis; PSI, patient-specific instrumentation; VAS, visual analog scale; AOFAS, American Orthopedic Foot and Ankle Society; SF-36, Short Form Survey; PROM, patient-reported outcome measure; AOS, ankle osteoarthritis scale; PROMIS, Patient-Reported Outcomes Measurement Information System; FAOS, foot and ankle outcome score.

**Table 3:** Literature review of articles on 3D printing in arthrodesis.

Author	Published Year	Study Design	No. of Cases	Ankle Pathology	Surgery Type	Outcomes	Mean Follow-Up Period (Months)	3D Printing Use
Hsu et al. [57]	2015	Case report	1	Persistent distal tibial nonunion with large bony defect	TTC arthrodesis	Minimal pain, ambulation, and work independency, no wound complication	12	Customized implant
Steele et al. [58]	2020	Retrospective	8 (3D spherical implant), 7 (femoral head allograft)	Severe ankle bone defects	TTC arthrodesis	The proportion of total fused articulations and the number of patients who successfully fused all three articulations were notably higher in the 3D sphere group. In contrast, the rate of graft resorption was significantly elevated in the femoral head allograft group.	23, 30	Customized implant
Lorena et al. [59]	2020	Retrospective	7	Ankle OA with segmental bone defects	TTC arthrodesis	Participants reported performing daily activities without pain. Both the AOFAS score and VAS showed significant improvement. Six patients achieved over 50% bony bridging, while one patient underwent below-knee amputation due to a recurrence of chronic osteomyelitis.	21	Customized implant
Eamon et al. [60]	2021	Retrospective	3	End-stage talar AVN	TTC arthrodesis	AOFAS score (improved), radiographs (satisfactory radiological union)	32	Customized implant
Antounian et al. [61]	2024	Case report	1	Post-traumatic talar AVN and failed ankle arthrodesis	TTC arthrodesis	Patients could walk without canes or crutches and did so without pain. Radiographs indicated that the custom-designed implant fit the bony defect area.	24	Customized implant
Kim et al. [62]	2024	Retrospective	113	Charcot arthropathy or end-stage ankle OA	TTC arthrodesis	Mean NRS pain (improved from 6.6 to 2.0), 11 patients were able to ambulate independently	44.6	Customized implant
Duan et al. [63]	2018	Retrospective	15 (PSI), 14 (control)	Ankle OA	Ankle arthrodesis (arthroscopic)	The duration required to drill the Kirschner wires into the correct position was notably shorter in the PSI group. There were no significant differences in fusion time or AOFAS scores, and neither groups experienced obvious complications.	25.2	PSI (guide)
Liang et al. [64]	2022	Retrospective	13	Distal tibia tumor (wide or marginal resection)	Ankle arthrodesis	Mean MSTS-93 score (28.0 ± 1.5), 1 case of periprosthetic infection after paronychia	26.8	Customized implant
Duan et al. [65]	2019	Retrospective	14 (PSI), 16 (control)	Subtalar OA	Subtalar arthrodesis	The duration for positioning the Kirschner wires was notably shorter in the PSI group. There were two instances of re-drilling in the PSI group compared to eight in the control group. The AOFAS scores showed no significant differences, and radiographic fusion was confirmed in all cases.	24	PSI (guide)

TTC, tibiotalocalcaneal; 3D, three-dimensional; OA, osteoarthritis; AOFAS, American Orthopedic Foot and Ankle Society; VAS, visual analog scale; AVN, avascular necrosis; NRS, numeric rating scale; PSI, patient-specific instrumentation; MSTS, Musculoskeletal Tumor Society.

**Table 4:** Literature review of articles on 3D printing in supramalleolar osteotomy.

Author	Published Year	Study Design	No. of Cases	Ankle Pathology	Surgery Type	Outcomes	Mean Follow-Up Period (Months)	3D Printing Use
Faict et al. [71]	2021	Retrospective	5	Ankle OA	SMO	EFAS score, FAOS, and VAS (significantly improved), radiographs (healing of the osteotomy site was confirmed on WBCT), radiographic parameters (improvement of the TAS, tibiotalar angle, and hindfoot angle)	40.8	PSI (guide)
Wang et al. [73]	2022	Retrospective	11 (PSI), 17 (control)	Ankle OA	SMO	Mean operating time, postoperative hospital stay, number of fluoroscopy examinations, and albumin reduction (significantly lower in the PSI group compared to the control group)	33.4	PSI (guide plate)
Zhang et al. [72]	2022	Retrospective	7 (PSI), 9 (control)	Ankle OA	SMO	Mean operating time, intraoperative blood loss, and intraoperative fluoroscopy time (significantly lower in the PSI group compared to the control group)	13.9	Customized implant (cage), PSI (guide)

OA, osteoarthritis; SMO, supramalleolar osteotomy; EFAS, European Foot and Ankle Society; FAOS, foot and ankle outcome score; VAS, visual analog scale; WBCT, weight-bearing computed tomography; TAS, tibial anterior surface angle; PSI, patient-specific instrumentation.

## Data Availability

Not applicable.

## Abbreviations

3D, three-dimensional; TTR, total talus replacement; AVN, avascular necrosis; TTC, tibiotalocalcaneal; TAA, total ankle arthroplasty; SMO, supramalleolar osteotomy; TTNR, total talonavicular replacement; BK, below-knee; TTR, total talar replacement; ROM, range of motion; CT, computed tomography; OA, osteoarthritis; MSTS, Musculoskeletal Tumor Society score; AOFAS, American Orthopedic Foot and Ankle Society; DF, dorsiflexion; PF, plantarflexion; VAS, visual analog scale; SF-36, Short Form-36 Health Survey; PSI, patient-specific instrumentation; PROM, patient-reported outcome measure; PROMIS, Patient-Reported Outcomes Measurement Information System; WBCT, weight-bearing CT; TAS, tibial anterior surface angle
